# PPSP: prediction of PK-specific phosphorylation site with Bayesian decision theory

**DOI:** 10.1186/1471-2105-7-163

**Published:** 2006-03-20

**Authors:** Yu Xue, Ao Li, Lirong Wang, Huanqing Feng, Xuebiao Yao

**Affiliations:** 1 School of Life Science, University of Science and Technology of China, Hefei, Anhui, 230027, China; 2 Department of Electronic Science and Technology, University of Science and Technology of China, Hefei, Anhui, 230027, China; 3 Department of Physiology, Morehouse School of Medicine, Atlanta, GA 30310, USA

## Abstract

**Background:**

As a reversible and dynamic post-translational modification (PTM) of proteins, phosphorylation plays essential regulatory roles in a broad spectrum of the biological processes. Although many studies have been contributed on the molecular mechanism of phosphorylation dynamics, the intrinsic feature of substrates specificity is still elusive and remains to be delineated.

**Results:**

In this work, we present a novel, versatile and comprehensive program, PPSP (Prediction of PK-specific Phosphorylation site), deployed with approach of Bayesian decision theory (BDT). PPSP could predict the potential phosphorylation sites accurately for ~70 PK (Protein Kinase) groups. Compared with four existing tools Scansite, NetPhosK, KinasePhos and GPS, PPSP is more accurate and powerful than these tools. Moreover, PPSP also provides the prediction for many novel PKs, say, TRK, mTOR, SyK and MET/RON, etc. The accuracy of these novel PKs are also satisfying.

**Conclusion:**

Taken together, we propose that PPSP could be a potentially powerful tool for the experimentalists who are focusing on phosphorylation substrates with their PK-specific sites identification. Moreover, the BDT strategy could also be a ubiquitous approach for PTMs, such as sumoylation and ubiquitination, etc.

## Background

Protein phosphorylation, as one of the most common post-translational modifications (PTM), is reversibly and transiently performed by protein kinases (PKs). It plays crucial regulatory roles in a variety of biological cellular processes, including transcription [1], translation [2], mitosis/cell cycle [3], neurite outgrowth [4,5] and signal transductions [6], etc. Many previous researches have contributed to increase our knowledge on phosphorylation. However, the intrinsic features of phosphorylation dynamics are still cryptic and remain to be dissected. Biochemically, the catalytic site of a PK hydrolyzes adenosine triphosphate (ATP) and transfers a phosphate moiety to the acceptor residue (S/T, Y in eukaryotes) in the substrate. Each PK only modifies a defined subset of substrates specifically to ensure signaling fidelity, and defects of PK function often induce a variety of diseases and cancers [7].

There is an extensively-adopted hypothesis that PKs phosphorylate their substrates at the specific sites (consensus sequence) flanking with canonical motif [8-10]. To date, the consensus motifs of ~30 PKs have been reported [11]. However, there is still a large number of PKs with their specific target motifs remained to be identified. Therefore, elucidating PK-specific phosphorylation sites on the substrates is the foundation of understanding the molecular mechanism of substrates specificity and important for the biomedical drug design. However, it has been described that only consensus motif is not enough for providing the specificity of PK recognition *in vivo *[12]. There are numerous mechanisms have been proposed to contribute specificity for PKs, such as co-complex of PKs with their substrates, subcellular co-localization, interacting through modular docking sites, phosphopeptide-binding mechanisms, etc [12-17]. In a cell, protein kinase usually forms a tight complex with its target either through a third scaffold protein, or by recognizing and binding short sequence of the substrate, known as a docking site [12,18]. Moreover, phosphopeptide-binding domains (PBDs) are also important to achieve substrate specificity. Numerous PBDs (PTB, WW, SH2, SH3, FHA, MH2, WD40, Polo-box, and 14-3-3, etc) bind the phosphorylated forms of specific proteins, with recognizing distinct peptides surrounding the phosphorylated sites (pS/T, or pY) [14-17,19]. However, how these mechanisms achieve the additional specificity for PKs beyond phosphorylated motifs is still elusive, and there are very few computational studies published on this area [13,16,19]. In addition, many docking sites and PBDs still remain to be dissected. Thus, in this work, we focus on the prediction of PK-specific phosphorylation sites based on profiles/features of the surrounding primary sequences, as previously described [8-10].

Conventional experimental identifications of PK-specific phosphorylation sites on substrates *in vivo *and *in vitro *have provided the foundation of understanding the mechanisms of phosphorylation dynamics. However, these experiments are often time-consuming and expensive. And the enzymatic activity of the PKs are usually diminished or impeded *in vitro*, hampering on the studies of phosphorylation greatly. Recently, phospho-proteomic studies with mass spectrometry (MS) approaches have generated numerous data in yeast [20], mouse [21], and human [8], etc. But in these cases, it's still difficult to distinguish the PK-specific sites on the substrates. With regard of this, it is of note that the *in silico *prediction of PK-specific phosphorylation sites is in urgent need for the further experimental manipulation. To address this question, several excellent predictors have been implemented and reported [13,22-25]. For example, NetPhos has employed the consensus-motif-based approaches implemented in the artificial neural networks (ANNs) algorithm [22]. The enhanced version, NetPhosK can predict PK-specific phosphorylation sites for ~17 PKs [23]. Another online tool Scansite [13] has constructed the motif profiles of phosphorylation sites for ~20 PKs, and could predict their target sites, respectively. Previously, we have reported a web server GPS, which has been implemented in GPS (Group-based phosphorylation Predicting and Scoring) algorithm [26,27]. GPS could predict ~70 kinds of PK-specific phosphorylation sites, and gain excellent performance on several PK groups, especially for kinase Aurora-B. Recently, a novel and excellent web tool of KinasePhos has been incorporated with HMM (Hidden Markov Models) algorithm and constructed for phosphorylation sites predicting of 18 PK-specific groups [24,25].

In this study, we present a novel, convenient and comprehensive program, PPSP (Prediction of PK-specific Phosphorylation site), implemented in an algorithm of Bayesian decision theory (BDT). An online PPSP web service has been also constructed, accurately predicting PK-specific phosphorylation sites for 68 PK groups. The prediction performances of PPSP are satisfactory on several well-studied PKs and comparable with the other existing tools NetPhosK, Scansite, KinasePhos and GPS. Moreover, PPSP also provides the accurate prediction for many novel PKs, such as TRK, mTOR, SyK, and MET/RON, etc. Obviously, PPSP is more accurate and powerful. Therefore, we propose that PPSP could be useful and insightful for further experimental design. In addition, the prediction results of PPSP combined with delicate experiments verifications will propel our understanding of the mechanisms of phosphorylation into a new phase.

## Implementation

### Preparation of training data set

Firstly, we obtained the data set of phosphorylation sites from Phospho.ELM (Ver 2.0, Sep. 2004) [28] and filtered the phosphorylation sites without information of PKs. There were ~1,400 sites preserved. We also manually curated the recent literature and acquired ~660 items (Before Nov. 2004). These newly curated data has been submitted to Phospho.ELM for further integration. The two data sets were integrated, and the redundant items were removed if two items exactly pinned point to the same phosphorylation site from one protein sequence. Then the total training data set contained >2,000 non-redundant positive data with very few homologous sites (see additional file 1).

Since there were several PKs with too few known phosphorylation sites, we clustered them into distinct sub-groups based on sequence homology. For example, eight ribosomal protein S6 kinases (RSK1, Q15418; RSK3, Q15349; RSK2, P51812; MSK1, O75676; MSK1, O75582; RSK4, Q9UK32; S6K1, P23443; STK14B, Q9UBS0) are homologous with high similarity, so we clustered these PKs into a unique PK group of S6K (Ribosomal protein S6 kinase, or RSK). In total, we have enabled 68 PK grouped.

Although Swiss-Prot also curates a huge amount of phosphorylation sites, we have found ~69% of the annotation to be ambiguous (7,924 of 11,520) (see additional file 2). There are only 842 items to be kinase-specific sites, and only 18 PKs with not less than ten sites (see additional file 3). Phospho.ELM has been constructed based on the rationale of allowing both experimentalists and bioinformatists to easily access extensive information of phosphoproteins with their sites, i.e., tracking the primary reference to find whether the site is really phosphorylated, identified *in vivo *or *in vitro*, and the relationship between the phosphorylation with physiological response [28]. And these data has been collected from literature manually with high quality. Taken together, although other resources also have collected some phosphorylation sites, we chose Phospho.ELM for its comprehensiveness.

### Positive & negative control for evaluation

The sequence information of these phosphorylation substrates was retrieved from ExPASy. As previously described [11], we adopted the experimental phosphorylation sites as the positive control, while all other residues (S/T or Y) in the phosphorylation substrates were regarded as the negative control. The detailed statistics of the positive and negative data sets categorized by PK groups is available (see additional file 4).

#### Bayesian Decision Theory (BDT)

Supposed that we have an unclassified data *x *that belongs to one of two certain categories: *C1 *(defined as phosphorylated sites in this work) and *C2 *(defined as non-phosphorylated sites). In addition, suppose the posterior probability of x for these two categories can be denoted as: *p*(*C*_1_|*x*) and *p*(*C*_2_|*x*). Then the probability of wrong prediction is:

P(error|x)=p(C1|x),if x∈C2p(C2|x),if x∈C1     (1)
 MathType@MTEF@5@5@+=feaafiart1ev1aaatCvAUfKttLearuWrP9MDH5MBPbIqV92AaeXatLxBI9gBaebbnrfifHhDYfgasaacH8akY=wiFfYdH8Gipec8Eeeu0xXdbba9frFj0=OqFfea0dXdd9vqai=hGuQ8kuc9pgc9s8qqaq=dirpe0xb9q8qiLsFr0=vr0=vr0dc8meaabaqaciaacaGaaeqabaqabeGadaaakeaacqWGqbaucqGGOaakcqWGLbqzcqWGYbGCcqWGYbGCcqWGVbWBcqWGYbGCcqGG8baFcqWG4baEcqGGPaqkcqGH9aqpfaqabeGabaaabaGaemiCaaNaeiikaGIaem4qam0aaSbaaSqaaiabigdaXaqabaGccqGG8baFcqWG4baEcqGGPaqkcqGGSaalcqWGPbqAcqWGMbGzcqqGGaaicqWG4baEcqGHiiIZcqWGdbWqdaWgaaWcbaGaeGOmaidabeaaaOqaaiabdchaWjabcIcaOiabdoeadnaaBaaaleaacqaIYaGmaeqaaOGaeiiFaWNaemiEaGNaeiykaKIaeiilaWIaemyAaKMaemOzayMaeeiiaaIaemiEaGNaeyicI4Saem4qam0aaSbaaSqaaiabigdaXaqabaaaaOGaaCzcaiaaxMaadaqadaqaaiabigdaXaGaayjkaiaawMcaaaaa@61F9@

To minimize the expectation of error probability that is defined as [29]:

*P*(*error*) = ∫*P*(*error*|*x*)*p*(*x*)*dx *   (2)

It is obvious that one should choose the more probable category as the prediction result, which can be formulated by the Bayesian Decision Rule [29]:

predict x as{C1,if P(C1|x)>P(C2|x)C2,otherwise     (3)
 MathType@MTEF@5@5@+=feaafiart1ev1aaatCvAUfKttLearuWrP9MDH5MBPbIqV92AaeXatLxBI9gBaebbnrfifHhDYfgasaacH8akY=wiFfYdH8Gipec8Eeeu0xXdbba9frFj0=OqFfea0dXdd9vqai=hGuQ8kuc9pgc9s8qqaq=dirpe0xb9q8qiLsFr0=vr0=vr0dc8meaabaqaciaacaGaaeqabaqabeGadaaakeaacqWGWbaCcqWGYbGCcqWGLbqzcqWGKbazcqWGPbqAcqWGJbWycqWG0baDcaaMc8UaemiEaGNaaGPaVlabdggaHjabdohaZnaaceaabaqbaeaabiGaaaqaaiabdoeadnaaBaaaleaacqaIXaqmaeqaaOGaeiilaWcabaGaemyAaKMaemOzayMaaGPaVlabdcfaqjabcIcaOiabdoeadnaaBaaaleaacqaIXaqmaeqaaOGaeiiFaWNaemiEaGNaeiykaKIaeyOpa4JaemiuaaLaeiikaGIaem4qam0aaSbaaSqaaiabikdaYaqabaGccqGG8baFcqWG4baEcqGGPaqkaeaacqWGdbWqdaWgaaWcbaGaeGOmaidabeaakiabcYcaSaqaaiabd+gaVjabdsha0jabdIgaOjabdwgaLjabdkhaYjabdEha3jabdMgaPjabdohaZjabdwgaLbaaaiaawUhaaiaaxMaacaWLjaWaaeWaaeaacqaIZaWmaiaawIcacaGLPaaaaaa@6A7C@

Furthermore, by definition we can introduce the loss function λ(α_*i*_|*C*_*j*_), where α_*i*_,*i *= 1,2 is the finite set of possible solution. Thus the expected loss (risk) of taking action α_*i *_is:

R(αi|x)=∑l=12λ(αi|Cl)P(Cl|x)     (4)
 MathType@MTEF@5@5@+=feaafiart1ev1aaatCvAUfKttLearuWrP9MDH5MBPbIqV92AaeXatLxBI9gBaebbnrfifHhDYfgasaacH8akY=wiFfYdH8Gipec8Eeeu0xXdbba9frFj0=OqFfea0dXdd9vqai=hGuQ8kuc9pgc9s8qqaq=dirpe0xb9q8qiLsFr0=vr0=vr0dc8meaabaqaciaacaGaaeqabaqabeGadaaakeaacqWGsbGucqGGOaakcqaHXoqydaWgaaWcbaGaemyAaKgabeaakiabcYha8jabdIha4jabcMcaPiabg2da9maaqahabaGaeq4UdWMaeiikaGIaeqySde2aaSbaaSqaaiabdMgaPbqabaGccqGG8baFcqWGdbWqdaWgaaWcbaGaemiBaWgabeaakiabcMcaPiabdcfaqjabcIcaOiabdoeadnaaBaaaleaacqWGSbaBaeqaaOGaeiiFaWNaemiEaGNaeiykaKcaleaacqWGSbaBcqGH9aqpcqaIXaqmaeaacqaIYaGma0GaeyyeIuoakiaaxMaacaWLjaWaaeWaaeaacqaI0aanaiaawIcacaGLPaaaaaa@5448@

In this condition, the goal of optimization becomes to minimize the overall risk for every *x*. Similar to the rationale of Bayesian Decision Rule, we can obtain the best performance by computing *R*(α_*i*_|*x*) for each solution α_*i *_and choose that for which has the minimal overall risk (also named as Bayes Risk) [29].

### Training and prediction procedure

In this study, a local ennea-peptide (9aa) is deployed to define a candidate phosphorylation site, which has 4 upstream and 4 downstream residues of the potential phosphorylation site and can be denoted as x→
 MathType@MTEF@5@5@+=feaafiart1ev1aaatCvAUfKttLearuWrP9MDH5MBPbIqV92AaeXatLxBI9gBaebbnrfifHhDYfgasaacH8akY=wiFfYdH8Gipec8Eeeu0xXdbba9frFj0=OqFfea0dXdd9vqai=hGuQ8kuc9pgc9s8qqaq=dirpe0xb9q8qiLsFr0=vr0=vr0dc8meaabaqaciaacaGaaeqabaqabeGadaaakeaacuWG4baEgaWcaaaa@2E37@ = (*x*_1_,*x*_2_,...,*x*_9_)'. Given some positive (training) data, there are many ways to estimate *R*(α_*i*_|*x*) (where α_1 _and α_2 _denote different prediction results: true and false phosphorylation sites, respectively). One simple way is to assume that all flanking residues are mutual independent, and then the Bayes Risk can be formulated as:

R(αi|x→)=∑j=19R(αi|xj)     (5)
 MathType@MTEF@5@5@+=feaafiart1ev1aaatCvAUfKttLearuWrP9MDH5MBPbIqV92AaeXatLxBI9gBaebbnrfifHhDYfgasaacH8akY=wiFfYdH8Gipec8Eeeu0xXdbba9frFj0=OqFfea0dXdd9vqai=hGuQ8kuc9pgc9s8qqaq=dirpe0xb9q8qiLsFr0=vr0=vr0dc8meaabaqaciaacaGaaeqabaqabeGadaaakeaacqWGsbGucqGGOaakcqaHXoqydaWgaaWcbaGaemyAaKgabeaakiabcYha8jqbdIha4zaalaGaeiykaKIaeyypa0ZaaabCaeaacqWGsbGucqGGOaakcqaHXoqydaWgaaWcbaGaemyAaKgabeaakiabcYha8jabdIha4naaBaaaleaacqWGQbGAaeqaaOGaeiykaKcaleaacqWGQbGAcqGH9aqpcqaIXaqmaeaacqaI5aqoa0GaeyyeIuoakiaaxMaacaWLjaWaaeWaaeaacqaI1aqnaiaawIcacaGLPaaaaaa@4BCB@

R(αi|xj)=E(λ|xj,αi)=∑l=12∑k=120λ(j,k|αi,Cl)p(Cl|xj)     (6)
 MathType@MTEF@5@5@+=feaafiart1ev1aaatCvAUfKttLearuWrP9MDH5MBPbIqV92AaeXatLxBI9gBaebbnrfifHhDYfgasaacH8akY=wiFfYdH8Gipec8Eeeu0xXdbba9frFj0=OqFfea0dXdd9vqai=hGuQ8kuc9pgc9s8qqaq=dirpe0xb9q8qiLsFr0=vr0=vr0dc8meaabaqaciaacaGaaeqabaqabeGadaaakeaacqWGsbGucqGGOaakcqaHXoqydaWgaaWcbaGaemyAaKgabeaakiabcYha8jabdIha4naaBaaaleaacqWGQbGAaeqaaOGaeiykaKIaeyypa0JaemyrauKaeiikaGIaeq4UdWMaeiiFaWNaemiEaG3aaSbaaSqaaiabdQgaQbqabaGccqGGSaalcqaHXoqydaWgaaWcbaGaemyAaKgabeaakiabcMcaPiabg2da9maaqahabaWaaabCaeaacqaH7oaBcqGGOaakcqWGQbGAcqGGSaalcqWGRbWAcqGG8baFcqaHXoqydaWgaaWcbaGaemyAaKgabeaakiabcYcaSiabdoeadnaaBaaaleaacqWGSbaBaeqaaOGaeiykaKIaemiCaaNaeiikaGIaem4qam0aaSbaaSqaaiabdYgaSbqabaGccqGG8baFcqWG4baEdaWgaaWcbaGaemOAaOgabeaakiabcMcaPaWcbaGaem4AaSMaeyypa0JaeGymaedabaGaeGOmaiJaeGimaadaniabggHiLdaaleaacqWGSbaBcqGH9aqpcqaIXaqmaeaacqaIYaGma0GaeyyeIuoakiaaxMaacaWLjaWaaeWaaeaacqaI2aGnaiaawIcacaGLPaaaaaa@71C0@

Here *p*(*C*_*l*_|*x*_*j*_) is the posterior probability of *x*_*j *_belonging to category *C*_*l *_and can be further described by the Bayesian formula:

p(Cl|xj)=p(xj|Cl)p(Cl)p(xj)=p(xj|Cl)p(Cl)∑l=12p(xj|Cl)p(Cl),l=1,2     (7)
 MathType@MTEF@5@5@+=feaafiart1ev1aaatCvAUfKttLearuWrP9MDH5MBPbIqV92AaeXatLxBI9gBaebbnrfifHhDYfgasaacH8akY=wiFfYdH8Gipec8Eeeu0xXdbba9frFj0=OqFfea0dXdd9vqai=hGuQ8kuc9pgc9s8qqaq=dirpe0xb9q8qiLsFr0=vr0=vr0dc8meaabaqaciaacaGaaeqabaqabeGadaaakeaacqWGWbaCcqGGOaakcqWGdbWqdaWgaaWcbaGaemiBaWgabeaakiabcYha8jabdIha4naaBaaaleaacqWGQbGAaeqaaOGaeiykaKIaeyypa0ZaaSaaaeaacqWGWbaCcqGGOaakcqWG4baEdaWgaaWcbaGaemOAaOgabeaakiabcYha8jabdoeadnaaBaaaleaacqWGSbaBaeqaaOGaeiykaKIaemiCaaNaeiikaGIaem4qam0aaSbaaSqaaiabdYgaSbqabaGccqGGPaqkaeaacqWGWbaCcqGGOaakcqWG4baEdaWgaaWcbaGaemOAaOgabeaakiabcMcaPaaacqGH9aqpdaWcaaqaaiabdchaWjabcIcaOiabdIha4naaBaaaleaacqWGQbGAaeqaaOGaeiiFaWNaem4qam0aaSbaaSqaaiabdYgaSbqabaGccqGGPaqkcqWGWbaCcqGGOaakcqWGdbWqdaWgaaWcbaGaemiBaWgabeaakiabcMcaPaqaamaaqahabaGaemiCaahaleaacqWGSbaBcqGH9aqpcqaIXaqmaeaacqaIYaGma0GaeyyeIuoakiabcIcaOiabdIha4naaBaaaleaacqWGQbGAaeqaaOGaeiiFaWNaem4qam0aaSbaaSqaaiabdYgaSbqabaGccqGGPaqkcqWGWbaCcqGGOaakcqWGdbWqdaWgaaWcbaGaemiBaWgabeaakiabcMcaPaaacqGGSaalcqWGSbaBcqGH9aqpcqaIXaqmcqGGSaalcqaIYaGmcaWLjaGaaCzcamaabmaabaGaeG4naCdacaGLOaGaayzkaaaaaa@7FDD@

Here *p*(*C*_*l*_) is the prior probability of category *C*_*l *_and *p*(*x*_*j*_|*C*_*l*_) can be estimated by observing the occurrence of each residue in training data given the hypothesis of equation (5). Although there are much more false phosphorylation site in data set, we give equal prior probability for each category (no prior information), which can avoid bias prediction results. The loss function we construct is based on BLOSUM62 matrix [30] by considering the biochemical difference of residues, which can be formulated as:

λ(j,k|αi,Cl)={−BLOSUM62(j,k),if αi≠Cl0,if αi=Cl     (8)
 MathType@MTEF@5@5@+=feaafiart1ev1aaatCvAUfKttLearuWrP9MDH5MBPbIqV92AaeXatLxBI9gBaebbnrfifHhDYfgasaacH8akY=wiFfYdH8Gipec8Eeeu0xXdbba9frFj0=OqFfea0dXdd9vqai=hGuQ8kuc9pgc9s8qqaq=dirpe0xb9q8qiLsFr0=vr0=vr0dc8meaabaqaciaacaGaaeqabaqabeGadaaakeaacqaH7oaBcqGGOaakcqWGQbGAcqGGSaalcqWGRbWAcqGG8baFcqaHXoqydaWgaaWcbaGaemyAaKgabeaakiabcYcaSiabdoeadnaaBaaaleaacqWGSbaBaeqaaOGaeiykaKIaeyypa0ZaaiqaaeaafaqabeGacaaabaGaeyOeI0IaemOqaiKaemitaWKaem4ta8Kaem4uamLaemyvauLaemyta0KaeGOnayJaeGOmaiJaeiikaGIaemOAaOMaeiilaWIaem4AaSMaeiykaKIaeiilaWcabaGaemyAaKMaemOzayMaaGPaVlabeg7aHnaaBaaaleaacqWGPbqAaeqaaOGaeyiyIKRaem4qam0aaSbaaSqaaiabdYgaSbqabaaakeaacqaIWaamcqGGSaalaeaacqWGPbqAcqWGMbGzcaaMc8UaeqySde2aaSbaaSqaaiabdMgaPbqabaGccqGH9aqpcqWGdbWqdaWgaaWcbaGaemiBaWgabeaaaaaakiaawUhaaiaaxMaacaWLjaWaaeWaaeaacqaI4aaoaiaawIcacaGLPaaaaaa@6A7D@

Although other matrices could be also adopted, the BLOSUM62 matrix is chosen in this work. Moreover, we introduce a trade-off threshold *b *as the only parameter in this method to control the performance for different categories. Thus the final Discriminant function for prediction is:

predict x→ as{C1,if R(α2|x→)−R(α1|x→)>bC2,otherwise     (9)
 MathType@MTEF@5@5@+=feaafiart1ev1aaatCvAUfKttLearuWrP9MDH5MBPbIqV92AaeXatLxBI9gBaebbnrfifHhDYfgasaacH8akY=wiFfYdH8Gipec8Eeeu0xXdbba9frFj0=OqFfea0dXdd9vqai=hGuQ8kuc9pgc9s8qqaq=dirpe0xb9q8qiLsFr0=vr0=vr0dc8meaabaqaciaacaGaaeqabaqabeGadaaakeaacqWGWbaCcqWGYbGCcqWGLbqzcqWGKbazcqWGPbqAcqWGJbWycqWG0baDcaaMc8UafmiEaGNbaSaacaaMc8UaemyyaeMaem4Cam3aaiqaaeaafaqaaeGacaaabaGaem4qam0aaSbaaSqaaiabigdaXaqabaGccqGGSaalaeaacqWGPbqAcqWGMbGzcaaMc8UaemOuaiLaeiikaGIaeqySde2aaSbaaSqaaiabikdaYaqabaGccqGG8baFcuWG4baEgaWcaiabcMcaPiabgkHiTiabdkfasjabcIcaOiabeg7aHnaaBaaaleaacqaIXaqmaeqaaOGaeiiFaWNafmiEaGNbaSaacqGGPaqkcqGH+aGpcqWGIbGyaeaacqWGdbWqdaWgaaWcbaGaeGOmaidabeaakiabcYcaSaqaaiabd+gaVjabdsha0jabdIgaOjabdwgaLjabdkhaYjabdEha3jabdMgaPjabdohaZjabdwgaLbaaaiaawUhaaiaaxMaacaWLjaWaaeWaaeaacqaI5aqoaiaawIcacaGLPaaaaaa@6E20@

**Figure 1:**
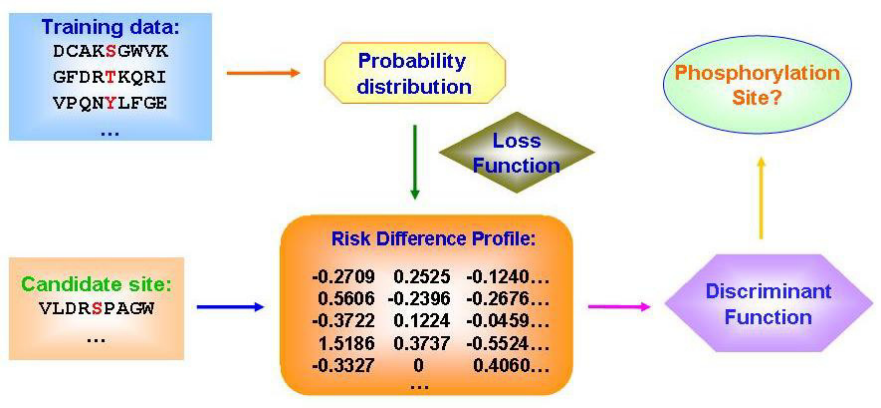
The outline of the training and procedure of PPSP.

The outline of the training and procedure in this work is illustrated in Figure 1. We first estimate the probability distribution of each residue of the true/false ennea-peptide within the training data. Then the Bayes risk for either potential solution (i.e true or false phosphorylation site) is calculated, respectively. To implement the final differential function in equation (9) effectively, we built a difference profile of Bayesian decision risk for each PK family/group in prediction. In this way, a candidate site for a given protein kinase is assessed in the profile and the outcome for each residue is summed up. If the difference of risks (false prediction minus true prediction) is greater than the threshold *b*, it will be predicted by PPSP as a negative site that can not be phosphorylated by this PK. Otherwise, PPSP will infer the site is as a potential phosphorylation site. In this work, the threshold for each PK has been optimized automatically.

## Results and discussions

### Prediction performance of PPSP

Three measurements, i.e., Sensitivity (*Sn*), Specificity (*Sp*), and Mathew correlation coefficient (*MCC*) are widely employed to evaluate the performance of prediction with definitions as below:

Sn=TPTP+FN,Sp=TNTN+FP,
 MathType@MTEF@5@5@+=feaafiart1ev1aaatCvAUfKttLearuWrP9MDH5MBPbIqV92AaeXatLxBI9gBaebbnrfifHhDYfgasaacH8akY=wiFfYdH8Gipec8Eeeu0xXdbba9frFj0=OqFfea0dXdd9vqai=hGuQ8kuc9pgc9s8qqaq=dirpe0xb9q8qiLsFr0=vr0=vr0dc8meaabaqaciaacaGaaeqabaqabeGadaaakeaafaqabeqacaaabaGaem4uamLaemOBa4Maeyypa0ZaaSaaaeaacqWGubavcqWGqbauaeaacqWGubavcqWGqbaucqGHRaWkcqWGgbGrcqWGobGtaaGaeiilaWcabaGaem4uamLaemiCaaNaeyypa0ZaaSaaaeaacqWGubavcqWGobGtaeaacqWGubavcqWGobGtcqGHRaWkcqWGgbGrcqWGqbauaaaaaiabcYcaSaaa@456D@

and

MCC=(TP×TN)−(FN×FP)(TP+FN)×(TN+FP)×(TP+FP)×(TN+FN).
 MathType@MTEF@5@5@+=feaafiart1ev1aaatCvAUfKttLearuWrP9MDH5MBPbIqV92AaeXatLxBI9gBaebbnrfifHhDYfgasaacH8akY=wiFfYdH8Gipec8Eeeu0xXdbba9frFj0=OqFfea0dXdd9vqai=hGuQ8kuc9pgc9s8qqaq=dirpe0xb9q8qiLsFr0=vr0=vr0dc8meaabaqaciaacaGaaeqabaqabeGadaaakeaacqWGnbqtcqWGdbWqcqWGdbWqcqGH9aqpdaWcaaqaaiabcIcaOiabdsfaujabdcfaqjabgEna0kabdsfaujabd6eaojabcMcaPiabgkHiTiabcIcaOiabdAeagjabd6eaojabgEna0kabdAeagjabdcfaqjabcMcaPaqaamaakaaabaGaeiikaGIaemivaqLaemiuaaLaey4kaSIaemOrayKaemOta4KaeiykaKIaey41aqRaeiikaGIaemivaqLaemOta4Kaey4kaSIaemOrayKaemiuaaLaeiykaKIaey41aqRaeiikaGIaemivaqLaemiuaaLaey4kaSIaemOrayKaemiuaaLaeiykaKIaey41aqRaeiikaGIaemivaqLaemOta4Kaey4kaSIaemOrayKaemOta4KaeiykaKcaleqaaaaakiabc6caUaaa@6698@

Among the data with positive predictions by PPSP, the real positives are regarded as *true positives *(*TP*), while the others are defined as *false positives *(*FP*). Among the data with negative predictions by PPSP, the real positives are regarded as *false negatives *(*FN*), while the others are defined as *true negatives *(*TN*). The Sensitivity (*Sn*) and Specificity (*Sp*) illustrate the correct prediction ratios of positive and negative data sets respectively. But when the number of positive data and negative data differ too much from each other, the Mathew correlation coefficient (*MCC*) should be calculated to assess the prediction performance. The value of *MCC *ranges from -1 to 1, and bigger *MCC *stands for better prediction performance.

To assess whether PPSP is unbiased and robust for prediction, we adopt the standard method "Jack-Knife" validation. We perform the Jack-Knife validation for these PKs by removing one real site from the training data set at a time and re-calculating the *Sn *&*Sp*, respectively. The final results are the average of the all *Sn *&*Sp *of the Jack-Knife validation. Although "Jack-Knife" validation does make sense when the size of the data set is small (i.e., N < 30), we have also taken an additional test with *n*-fold (4-, 6-, 8- and 10-fold in this work) cross-validation for 22 PK groups with larger positive data set (N ≥ 30). As previously proposed [25], the tests are repeated for 20 times and the *Sn *&*Sp *is computed each time. Then the average Sn & Sp are calculated as the final results (see additional file 5).

In table 1, we list the prediction performances for four most well-studied PKs of PKA (Protein kinase A), CK2 (Casein Kinase II), ATM (Ataxia telangiectasia mutated) and S6K (Ribosomal protein S6 kinase, or RSK). The prediction performances of self-consistency, Jack-knife validation and *n*-fold cross-validation has been provided. For PKA, CK2, ATM and S6K, the *Sn *&*Sp *of the self-consistency is 92.31% & 97.40%, 93.33% & 96.46%, 92.59% & 91.98%, and 89.47% & 95.90%, while the Jack-Knife validation is 90.11% & 90.46%, 83.21% & 88.44%, 86.05% & 91.89%, 92.86% & 91.05%, respectively. Interestingly, the performances of *n*-fold cross-validation are very similar and consistent with the results of the Jack-Knife validation. So the PPSP is quite robust and unbiased for these well-studied PKs. Moreover, PPSP could predict for several novel PKs (>30, see additional file 4). In Table 2, we choose four PKs, including TRK (Neurotrophic tyrosine kinase receptor), mTOR (Mammalian target of rapamycin), SyK (Spleen tyrosine kinase), and MET/RON (Hepatocyte growth factor receptor/Macrophage-stimulating protein receptor), which predictors for them are not available previously. Interestingly, the prediction performance of PPSP is also satisfying. And the Jack-knife validation proposes that the PPSP approach is also robust and unbiased for these novel PKs. The full content of the prediction performance of PPSP is available from PPSP website.

**Figure 2:**
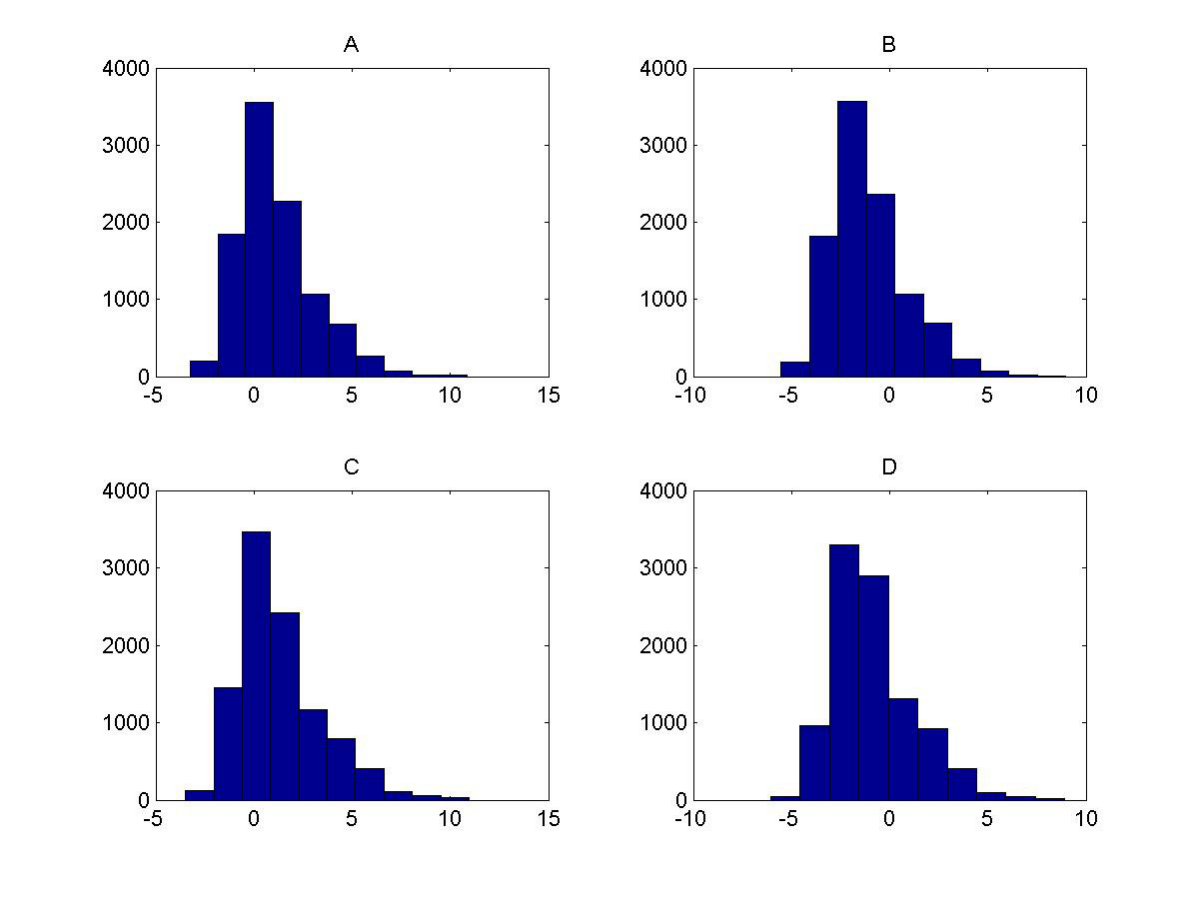
the distribution of risk difference of random and human proteome data set of PKA-specific site prediction is diagramed in Figure 2. A. Distribution of Risk Difference of random data set (serine) of PKA-specific site prediction. B. Distribution of Risk Difference of random data set (threonine) of PKA-specific site prediction. C. Distribution of Risk Difference of human proteome data set (serine) of PKA-specific site prediction. D. Distribution of Risk Difference of Human proteome data set (threonine) of PKA-specific site prediction.

To evaluate the performance of PPSP on the signal to noise for phosphorylation sites retrieval, we also perform two additional evaluations. Firstly, we randomly generate 10, 000 serine (S) and threonine (T) ennea-peptides for serine/threonine kinases (STKs), with 10, 000 tyrosine (Y) nona-peptides for tyrosine kinases (TKs). In addition, to determine the ability of the PPSP to retrieve potential real phosphorylation sites from the full proteome, we have downloaded the protein sequences of human proteome from public database . Again, we randomly retrieve 10, 000 S & T and Y ennea-peptides for STKs and TKs from the human proteome, respectively. Then we compute the Risk Difference (RD) of each ennea-peptide. Under the default threshold of PPSP, the percentile of the sites predicted to be potential true positive hits is listed (see in Table 3). The prediction results of random and human proteome data set are very similar. And the distribution of Risk Difference of random and human proteome data set of PKA-specific site prediction is diagramed in Figure 2. In this work, the default threshold of PKA is 3.5, and predicted Risk Differences of the most of the ennea-peptides from the two data sets are smaller than this cut-off. Based on these analyses, we propose that PPSP could efficiently predict the potential real sites with very low false positive hits. The ratio of Serine and Threonine is not exactly equal. However, we and others are unable to explain this question [25].

### Comparison of PPSP with Scansite, NetPhosK, KinasePhos and GPS

With four well-studied PKs of PKA, CK2, ATM and S6K as model kinases, we compare PPSP with four previous online prediction systems: Scansite, NetPhosK, KinasePhos and GPS. In Table 4, we list the prediction performances of Scansite, NetPhosK, KinasePhos and GPS for PKA, CK2, ATM and S6K, respectively. Since we can't re-perform the Jack-knife validation for the predictors, we submit the substrate sequence into these tools for prediction. And the self-consistency performance of PPSP is adopted here for comparison. Scansite has three thresholds for prediction, including high, medium and low stringency, while KinasePhos has paid attention to prediction specificity with three cut-off values, as 90%, 95% and 100%. And the default parameter is adopted for GPS. We calculate the prediction performances of Scansite and KinasePhos at three distinct thresholds, separately. As for NetPhosK, we only adopt the default cut-off value with 0.5, in mode of Prediction without filtering. Obviously, PPSP, NetPhosK, KinasePhos and GPS are better than Scansite. For PKA, the prediction performance of PPSP is 90.11% (*Sn*) and 91.70% (*Sp*), and outperforms to NetPhosK (*Sn *79.12% &*Sp *90.65%) with about 10% higher sensitivity and similar specificity. And for CK2, the performance of PPSP is 83.21% (*Sn*) and 90.01% (*Sp*), slightly higher than NetPhosK (*Sn *82.48% &*Sp *89.43%). The prediction performance of KinasePhos is similar with PPSP on PKA and CK2. However, for ATM, the NetPhosK is 86.01% (*Sn*) and 98.51% (*Sp*), whereas PPSP is 93.02% (*Sn*) and 94.06% (*Sp*). Although PPSP has a lower specificity than NetPhosK with ~4%, the sensitivity is high with ~7% enhanced. Finally, for S6K (also called as RSK in NetPhosK), although the specificity of PPSP (97.97%) and NetPhosK (97.14%) is quite similar, PPSP outperforms than NetPhosK with ~10% higher in sensitivity. With regard of this, we propose the prediction performance of PPSP could be at least comparable with the existing systems.

**Figure 3:**
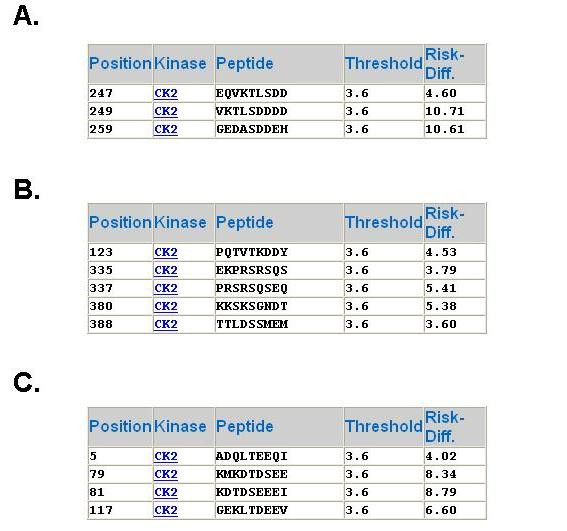
The prediction results of Bluetongue virus (BTV) nonstructural protein 2 (NS2), Drosophila transcription factor GAGA and human Calmodulin protein with PPSP. Figure 3A – prediction results of NS2; Figure 3B – prediction results of GAGA; Figure 3C – prediction results of Calmodulin.

However, the analysis and comparison above are only in theoretical and not intuitive. Furthermore, we browse the recent literature from PubMed and randomly choose some instances for comparison. One example is Bluetongue virus (BTV) nonstructural protein 2 (NS2, P23065), a substrate of CK2 [31]. As a nonspecific single-stranded RNA (ssRNA)-binding protein, NS2 accumulates in BTV-infected cells, and is functional in viral replication and morphogenesis [31-34]. NS2 could hydrolyze both ATP and GTP with high affinity, showing strong enzymatic activity [32]. Using mutagenesis analysis, CK2 was demonstrated to phosphorylate NS2 in two serine sites S249 and S259, probably modulating its RNA binding properties, enzymatic activity or influencing its ability to interact with other viral proteins [31]. For CK2-specific phosphorylation sites prediction, all of the four programs can detect them successfully (see in Table 5). In this case, the Scansite with medium stringency get the best hits. PPSP predict three sites as positive hits (T247, S249, and S259), but NetPhosK provide too much results with seven positive hits. Two additional instances are also provided in Table 5. One is *Drosophila *transcription factor protein GAGA (Q08605), regulating gene transcription and chromatin remodeling, etc [35]. The other is human Calmodulin protein (P62158) [36]. The prediction results of the four programs are shown in Table 5. And the online prediction of PPSP is diagramed in Figure 3. Obviously, for the well-studied PKs, i.e. CK2, PPSP is accurate and comparable with the existing tools.

### Application of PPSP to the novel PKs

For application of PPSP to the novel PKs, here we employ PPSP to predict the phosphorylation sites of TRK. TRK is a sub-family of receptor tyrosine kinases (RTK), consisting three highly similar homologs, TRK-A, -B, and -C [37]. TRK-A, -B, and -C could be activated specifically by nerve growth factor (NGF), brain-derived neurotrophic factor (BDNF) and NT-4/-5, and NT-3, respectively. Under activated state, TRK could regulate a variety of biological processes including cell survival, embryo, differentiation, proliferation, axon and dendrite growth and patterning, and apoptosis, etc [37].

**Figure 4:**
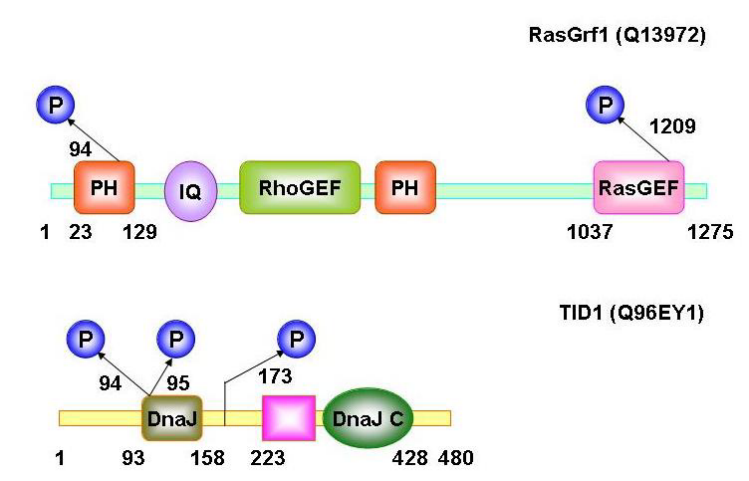
The diagram of potential phosphorylation sites of human RasGrf1 (Q13972) and TID1 (Q96EY1) by TRK.

Recently, protein Ras guanine-releasing factor 1 (RasGrf1, Q13972), a GTPase of the Ras and Rho family, has been proposed to be phosphorylated and interact with TRK-A, -B, -C in co-transfection experiments [5]. However, the exact phosphorylation sites of RasGrf1 by TRK remain to be identified. PPSP has predicted that there are totally two potential phosphorylation sites on RasGrf1 (Y94 & Y1209) (see in Figure 4). Moreover, the human tumorous imaginal disc 1 (TID1, Q96EY1) was verified as a substrate of TRK with co-immunoprecipitation (Co-IP) [4] and the phosphorylation sites were not elucidated. PPSP could predict that there are three candidate sites with Y94, Y95 and Y173 (see in Figure 4). These prediction results would be very useful for the further experimentation and elucidation phospho-regulation underlying cellular dynamics.

## Conclusion

In this work, we present a novel computational program–PPSP (prediction of PK-specific phosphorylation sites) based on Bayesian decision theory (BDT). We model a candidate phosphorylation motif as an ennea/nona-peptide (9aa) flanking with 4 upstream and 4 downstream residues of a potential phosphorylation site (S/T, or Y). With the BDT algorithm, we estimate the probability distributions of true and false phosphorylation sites and make prediction based on a loss function constructed with BLOSUM62 matrix [30]. We have evaluated the sensitivity and specificity of PPSP by "Jack-knife" validation. An online PPSP web service has been also constructed, accurately predicting PK-specific phosphorylation sites for 68 PK groups. For comparison with four reported systems Scansite, NetPhosK, KinasePhos and GPS, we take four well-studied PKs of PKA, CK2, ATM and S6K as model kinases. The prediction performances of PPSP are satisfactory judged using these well-studied PKs and comparable with the other existing tools. Moreover, PPSP also provides the accurate prediction for many novel PKs, such as TRK, mTOR, SyK, and MET/RON, etc. Thus, comparison with the previous work, PPSP provides more accurate and powerful ability. Moreover, the BDT approach could also be an extensive method for PTMs prediction, such as sumoylation and ubiquitination, etc. In addition, although many phospho-proteomic researches have generated numerous data [8,20,21], however, the up-regulated PKs still remain to be dissected. Despite the demonstration of phosphor-regulation of protein kinases and their respective substrates, the exact phosphorylation sites are unclear [4,5]. Taken together, the prediction results of PPSP should be insightful and important for further experiments. The combination of computational and experimental identifications will propel our understanding of phosphorylation dynamics into a new phase.

### Availability and requirements

PPSP has been implemented in Linux + Apache + PHP, and is freely available at: . A latest web browser (eg. Internet Explorer, Netscape, or Mozilla, etc) is required.

## Authors' contributions

YX and AL should be regarded as joint First Authors. YX and AL designed the methodology, carried out the analysis and drafted the manuscript. LW developed the web service, contributed several insightful opinions and improved manuscript greatly. XY coordinated the research and finalized the manuscript.

## Supplementary Material

Additional file 1 To test whether the training data sets are highly redundant, we retrieve all protein sequences for each PK-specific substrate. Then we use CD-HIT to check whether many protein sequences are highly homologous. The result of the eight PK groups employed in this work is listed. However, most of the PK-specific substrates are shown with low similarity. For CK2 and PKA, we carefully check each pairs of the homologous protein sequences. However, most of the phosphorylation sites are not homologous sites. Thus, we propose the training data set is proper for this work with low redundant. Click here for file

Additional file 2 The statistics of the annotations of the phosphorylation information from Swiss-Prot database. The entries annotated with "by similarity", "potential", "probable" or "partial" are regarded as ambiguous annotations. There are only 842 annotations of the kinase-specific phosphorylation sites provided. Click here for file

Additional file 3 The statistics of the annotations of the kinase-specific phosphorylation sites from Swiss-Prot database. There are only 18 PK groups with not less than ten sites. Click here for file

Additional file 4 Data set description for each protein kinase. Click here for file

Additional file 5 The prediction performance of PPSP (self-consistency, Jack-Knife validation and *n*-fold cross-validation) for 22 PK groups with large data set (N ≥ 30). Click here for file

## Figures and Tables

**Table 1:** The performances of self-consistency, Jack-knife validation and *n *-fold (4-, 6-, 8-, 10-fold in this work) cross-validation for four well-studied PKs of PKA, CK2, ATM and S6K. The *n*- fold cross-validation has been performed for the large data sets (N ≥ 30).

**PPSP**		**PKA**		**CK2**		**ATM**		**S6K**	
		*Sn*(%)	*Sp*(%)	*Sn*(%)	*Sp*(%)	*Sn*(%)	*Sp*(%)	*Sn*(%)	*Sp*(%)
**Self-consistency**		90.11	91.70	83.21	90.01	93.02	94.06	92.85	97.97
**Jack-knife**		90.11	90.46	83.21	88.44	86.05	91.89	92.86	91.05
	4-	90.11	90.43	81.02	87.90	86.37	90.14	N/A	N/A
***n*-fold cross-validation**	6-	90.11	90.52	81.02	88.34	86.37	90.60	N/A	N/A
	8-	90.11	90.45	81.75	88.48	86.05	90.65	N/A	N/A
	10-	90.11	90.48	81.75	88.22	86.05	91.39	N/A	N/A
**Data set (No.)**	**Positive**	173		142		43		14	
	**Negative**	8, 408		5, 332		2, 048		683	

**Table 2:** The self-consistency performance and Jack-knife validation for four novel PKs of TRK, mTOR, SyK and MET/RON.

**PPSP**		**TRK**		**mTOR**		**SyK**		**MET/RON**	
		*Sn*(%)	*Sp*(%)	*Sn*(%)	*Sp*(%)	*Sn*(%)	*Sp*(%)	*Sn*(%)	*Sp*(%)
**Self-consistency**		92.31	97.40	93.33	96.46	92.59	91.98	89.47	95.90
**Jack-knife**		84.62	96.10	93.33	91.27	77.79	86.79	73.68	91.80
**Data set**	**Positive**	13		14		27		19	
**(No.)**	**Negative**	77		433		251		122	

**Table 3:** With the default cut-off of PPSP, the percentile of the sites predicted to be potential true positive hits is listed. Both random ennea-peptides and data sets from human proteome have been computed, separately.

**PK group**	**Random ennea- peptides**			**Ennea-peptides from human proteome**		
	S	T	Y	S	T	Y
**PKA**	11.75%	2.20%		14.61%	3.20%	
**CK2**	9.18%	3.18%		12.60%	5.65%	
**ATM**	8.42%	1.96%		8.95%	2.13%	
**S6K**	14.72%	3.71%		14.90%	3.89%	
**mTOR**	5.95%	7.09%		8.20%	9.14%	
**TRK**			3.59%			3.94%
**Syk**			7.00%			9.74%
**Met/RON**			13.37%			13.77%

**Table 4:** The prediction performance of Scansite, NetPhosK, KinasePhos and GPS for four well-studied PKs of PKA, CK2, ATM and S6K.

	**PK Group**	**PKA**			**CK2**			**ATM**			**S6K**		
**Predictor**		*Sn*(%)	*Sp*(%)	*MCC*	*Sn*(%)	*Sp*(%)	*MCC*	*Sn*(%)	*Sp*(%)	*MCC*	*Sn*(%)	*Sp*(%)	*MCC*
**PPSP**	Default^a^	90.11	91.7	0.3841	83.21	90.01	0.3596	93.02	94.06	0.4627	92.85	97.97	0.6618
**ScanSite**	High^b^	21.98	99.96	0.4450	10.95	99.86	0.2655	18.6	99.8	0.3443	N/A	N/A	N/A
	Medium	44.51	99.39	0.5084	27.01	99.11	0.3342	25.58	98.89	0.2756	N/A	N/A	N/A
	Low	47.8	98.29	0.4041	54.02	96.34	0.3684	51.16	94.89	0.2739	N/A	N/A	N/A
**NetPhosK**	Default	79.12	90.65	0.3165	82.48	89.43	0.3464	86.01	98.51	0.6786	82.35	97.14	0.5404
**KinasePhos**	90% (Sp)^d^	90.72	91.3	0.3783	72.53	91.58	0.3384	88.37	87.8	0.3137	N/A	N/A	N/A
	95% (Sp)	89.18	94.62	0.4595	64.58	94.93	0.3806	88.37	92.14	0.3893	N/A	N/A	N/A
	100% (Sp)	76.8	98.47	0.6154	54.86	98.66	0.5222	86.05	96.89	0.5497	N/A	N/A	N/A
**GPS**	Default	88.88	90.57	0.3564	82.99	87.59	0.3210	90.86	89.55	0.3498	94.9	91.34	0.3964

a. The default parameters are employed for PPSP, NetPhosK and GPS.

b. ScanSite 2.0 has three thresholds for prediction, including high, medium and low stringencies.

c. N/A – not available.

d. KinasePhos has paid attention to prediction specificity with three cut-off values, as 90%, 95% and 100%.

**Table 5:** The experimental verified vs. predicted CK2-specific phosphorylation sites of Bluetongue virus (BTV) nonstructural protein 2 (NS2), Drosophila transcription factor GAGA and human Calmodulin protein.

**CK2**		**NS2 (P23065)**	**GAGA (Q08605) **	**Calmodulin (P62158)**
**Experimental Defined**		S249, S259	S378, S388	T79, S81, S101, T117
**PPSP**		T247, **S249**, **S259**	T123, S335, S337, S380, **S388**	T5, **T79**, **S81**, **T117**
**NetPhosK**		T87, T88, S204, T247, **S249**, **S259**, T266	**S388**, T394	T5, T28, T44, T62, **T79**, **S81**, **S101**, **T117**
	high	**S259**	N/A	N/A
**ScanSite**	medium	**S249**, **S259**	N/A	**S81**
	low	T88, S182, T247, **S249**, **S259**	T385, T394	**T79**, **S81**, **T117**
**KinasePhos**	90% (Sp)	T247, **S249**, **S259**	S240, S241, S339, **T378**, S386, S389, S391, S393, S397, S518, S521, S523	T5, T44,** T79**, **S81**, **S101**, **T117**
	95% (Sp)	**S249**, **S259**	S240, S339, **T378**, S386, S391, S397, S518, S521	T5, T44,** T79**, **S81**, **S101**, **T117**
	100% (Sp)	**S259**	S339, S521	T5, **T79**, **S81**, **S101**, **T117**
**GPS**		**S249**, **S259**	T123, S337, S339, S385, S386, **S388**, S518, S521	T5, T44, **T79**, **S81**, **T117**
